# Antibody display technologies from phages to cells: translational bottlenecks and AI-enabled opportunities

**DOI:** 10.3389/fbioe.2026.1789373

**Published:** 2026-05-26

**Authors:** Amrita Sahu, Punyatoya Das, Rohit Das, Indrajit Bhattacharya, Teeshyo Bhattacharya, Utpal Mohan, Remya Sreedhar, Somasundaram Arumugam

**Affiliations:** 1 National Institute of Pharmaceutical Education and Research, Kolkata, Kolkata, India; 2 Sister Nivedita University, Kolkata, India

**Keywords:** antibody library, artificial intelligence (AI), display technology, monoclonal antibody, phage

## Abstract

Beginning with the pioneering hybridoma technology developed in 1975, antibody generation methodologies have advanced substantially, culminating in today’s single-cell techniques. Each successive approach contributes unique applications, advantages, and drawbacks that reflect the field’s dynamic progress. We highlight the impact of integrating single-cell RNA sequencing (scRNA-seq) with display technologies. This holds potential for the healthcare industry by enabling efficient identification and development of diagnostic and therapeutic antibodies. Monoclonal antibodies (MAbs) produced via each major technology are discussed to illustrate practical outcomes. We have also explored the essential role of glycosylation in maintaining antibody stability and function. Furthermore, we discussed single-cell RNA sequencing (scRNA-seq) that enables high-resolution profiling of immune repertoires and tumour heterogeneity, facilitating the identification of antigen-specific antibodies and rare cell populations. Integration with microfluidics and computational analysis enhances biomarker discovery and cell-specific resolution. These advances support personalised therapies and accelerate next-generation antibody discovery. Finally, we address the emerging integration of machine learning and artificial intelligence in antibody discovery, emphasising recent advances in epitope mapping and predicting three-dimensional protein structures from primary amino acid sequences. Collectively, these developments are poised to revolutionise antibody engineering and expand its impact on therapeutic innovation.

## 1 Introduction

**FIGURE 1:**
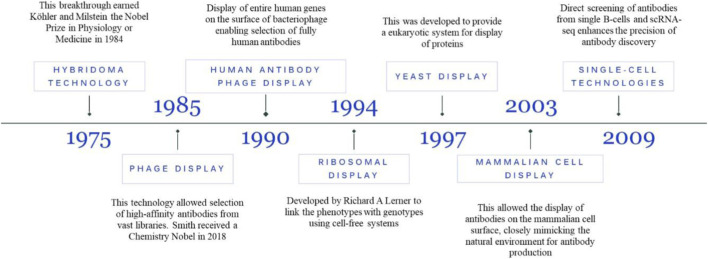
Timeline of key milestones in antibody discovery and display technologies. Schematic overview highlighting the evolution of antibody engineering platforms from hybridoma technology (1975), which enabled monoclonal antibody generation and earned the Nobel Prize, for the development of phage display (1985) and human antibody phage display (1990) for *in vitro* selection of fully human antibodies. Subsequent advances include ribosomal display (1994), a cell-free system linking genotype and phenotype; yeast surface display (1997), enabling eukaryotic folding and quantitative affinity screening; mammalian cell display (2003), allowing antibody expression and selection in a native post-translational environment; and single-cell technologies (2009), which permit direct antibody sequencing from individual B cells and have enhanced the precision and throughput of antibody discovery.

Antibodies are highly specific recognition molecules with exceptional target selectivity. Their architecture and diversity enable high specificity and affinity binding to a wide range of antigens. They are an essential tool in modern biotechnology. As of 2026, more than 50 years have passed since monoclonal antibodies (MAbs) were discovered using hybridoma technology. They have served as critical tools in disease diagnosis (Zuraw et al., 2023). Figure Figure1 illustrates the significant milestones and advancements in various display technologies to date. The FDA has approved numerous therapeutic antibodies in the last few years, and since then, the trend for MAbs has been on the rise. The number of antibody drugs in late-stage clinical trials increased to 178, representing strong growth compared to the 138 reported earlier (Crescioli et al., 2025).

Naked monospecific antibodies, antibody-drug conjugates (ADCs), bispecific/multispecific antibodies, immunoconjugates, antibody mixtures, and antibodies for radioimmunotherapy (including RICs and antibody-chelator conjugates) are various formats of antibodies optimized after the discovery phase. Hence, the discovery phase of antibodies is foundational for developing advanced antibody formats (Carter and Rajpal, 2022). MAbs have been revolutionary in creating tailor-made targeted therapy and have embodied the promise of precision medicine. Nevertheless, MAbs have been described as the fastest-growing class of therapeutic molecules (Singh et al., 2023). Display technologies are efficient biotechnological tools for developing target-specific antibodies for human use. They enable researchers to evaluate the therapeutic antibodies from large libraries (Bazan et al., 2012). This approach is beneficial for generating antibodies against poorly immunogenic or challenging targets and is widely applicable across diverse therapeutic areas, including infectious, autoimmune and allergic diseases. Antibodies developed via different display technologies have improved binding affinity and specificity compared with those derived from hybridoma technology (Bradbury et al., 2011). Figure 1 is a schematic overview highlighting the timeline of the evolution of antibody discovery techniques, which is discussed in detail below.

Unlike previous reviews that focus on individual antibody display platforms, this review provides a comparative, translational perspective by integrating multiple dimensions of antibody discovery. Specifically, it combines classical display technologies with emerging approaches such as single-cell RNA sequencing and artificial intelligence-driven design. As summarized in Table 1, the review highlights current limitations and bottlenecks, providing a more critical and application-oriented framework for next-generation antibody discovery.

**TABLE 1:** Comparative overview of major antibody display technologies.

Display technology	Library size (diversity)	PTM fidelity and folding	Screening speed and cost	Scalability and Chemistry, Manufacturing and controls (CMC) challenges	Key translational strengths	Major limitations
Phage display	10^10^–10^12^	No PTMs	Fast, low cost, highly automated	Excellent scalability; well-established good manufacturing practices (GMP) precedents for antibody discovery	Robust, cost-effective, ideal for *in vitro* affinity maturation	Limited PTM fidelity; potential developability issues
Bacterial display	10^8^–10^9^	Poor PTM fidelity; folding challenges	Fast, low cost	Scalable but limited translational relevance	Rapid screening and engineering	Misfolding of complex antibodies
Yeast surface display	10^9^–10^10^	Partial eukaryotic folding; limited PTMs	Moderate speed; medium cost	Scalable but less standardised for GMP	Quantitative affinity screening (FACS-based)	Smaller library size than phage; glycosylation differences
Ribosome display	10^12^–10^13^	No PTMs; folding challenges	Fast screening	Limited scalability; no direct GMP	Unmatched diversity; cell-free evolution	Protein instability; difficulty in translation to production stages
Mammalia n cell display	10^5^–10^7^	Full PTM fidelity; native IgG folding	Slow, expensive, labour-intensive	Scale-up challenges, direct relevance to GMP manufacturing	Highest physiological relevance	Cost, time, and limited diversity

The table summarizes key features, including library diversity, post-translational modifications (PTMs), screening speed and cost, scalability challenges, key translational strengths, and a few limitations of each technology.

## 2 Antibody discovery techniques

### 2.1 Hybridoma technology

In 1975, two scientists, Georges Kohler and Cesar Milstein, made a groundbreaking discovery by developing hybridoma technology, which enabled the immortalisation of antibody-producing cells and monoclonal antibodies highly antigen-specific. This approach eliminates the need for maintaining animals for antibody production compared to the previously produced old-fashioned polyclonal serum antibody (Mitra and Tomar, 2021). Antibody production via cell fusion enables the generation of highly specific antibodies against defined antigens. This approach also allowed the isolation of multiple hybrid cell lines that recognise the same antigen but differ in their antibody characteristics and effector functions, such as direct and indirect plaque formation (Köhler and Milstein, 1975). This technology involves fusing antigen-specific B cells with immortal myeloma cells (Pham, 2018). For the production of the desired antibody,the specific antigen is injected into the mice after a few weeks of immunization, B-cell differentiation takes place, which thereby leads to the activation of memory B-cells and plasma B-cells. The immune response is then evaluated by measuring antigen-specific antibody levels in serum using ELISA, and by analysing B-cell populations using flow cytometry (Pham, 2018; Mitra and Tomar, 2021). The animal is euthanised, and the spleen, bone marrow, or PBMCs are removed and centrifuged to collect activated B cells. After collection, B-cell fusion with myeloma cells occurs using polyethene glycol (PEG) or electrofusion. HAT (Hypoxanthine aminopterin thymidine) sensitive myeloma cells that lack the HGPRT (Hypoxanthine guanine phosphoribosyl transferase) genes are used for fusion. Using PEG as a promoter result in membrane fusion between B cells and myeloma cells, forming a heterokaryon. These fused cells are then selected from the media, while the dead/unfused cells are discarded (Greenfield, 2018). The newly fused cells are then cultured in HAT media for 10–14 days, during which nucleotide synthesis is blocked by aminopterin (in HAT), and hypoxanthine and thymidine are supplied, allowing the HGPRT-positive cells to survive. Over time, B cells die because their lifespan is limited, and unfused myeloma cells die because they lack HGPRT. The surviving cells are the hybridoma cells. Asingle hybridoma cell can be added to each well of a 96-well plate using the limited dilution method, or alternative methods such as flow cytometry sorting or semi-solid medium sorting methods. At the site of a specific epitope, a B-cell gene encodes an antibody in hybridoma cells, resulting in a monoclonal antibody. Hybridomas producing the desired antibodies are cultured either *in vivo* or *in vitro* using laboratory cell culture methods (Mitra and Tomar, 2021).

The first mAb approved for therapeutic use in humans was muromonab, which was murine and approved by the US FDA in 1986 (Liu, 2014). The first chimeric complete antibody approved for human use was rituximab, approved by the FDA in 1997. It targeted CD20 cells via various mechanisms, helping in the cure of on-Hodgkin lymphoma. It consists of human Fc constant regions and murine variable regions, with antigen-binding regions specific for CD20.

The specificity of Rituximab resides in the murine region, while the human Fc constant facilitates effective utilisation of complement and cell-mediated lysis mechanisms (Pescovitz, 2006). Another monoclonal antibody developed by hybridoma technology, I-tositumomab, was approved by the FDA in 2003. The antibody was conjugated to Iodine-131 and recognises and binds to the CD20 antigen, which is primarily expressed on B-lymphocytes. It initiates a host immune response against the B cells to which it is attached, and triggers apoptosis in a significant proportion of those cells (Findlay, 2007).

### 2.2 Display technologies

#### 2.2.1 Phage display

Phage display, discovered by Smith (1985), uses genetic engineering to produce antibodies against targets such as toxins, pathogens, and antigens (Smith, 1985). Foreign DNA fragments were introduced into gene III of filamentous bacteriophages, resulting in the expression of fusion proteins in which the inserted sequence is embedded within the phage coat protein. The fusion proteins were assembled into infectious virions that present the foreign peptide on the phage surface (Smith, 1985). John McCafferty’s lab was the first to demonstrate phage antibody display (Bazan et al., 2012). Phage antibody libraries have been used to select antibodies against infectious agents.

Antibody libraries used in display technologies can be broadly classified into natural, semi-synthetic, and fully synthetic libraries. Natural libraries are derived from immunised or naive B cells and reflect the *in vivo* immune repertoire. It can be done using human bone marrow, PBMCs and peripheral lymphoid tissues to isolate mRNA from cells and amplify the antibody-coding genes. In contrast, semi-synthetic libraries combine naturally derived antibody frameworks with synthetically diversified complementarity-determining regions (CDRs), thereby enhancing diversity while maintaining structural stability. Early semi-synthetic libraries, developed in the early 1990s, introduced targeted mutations into CDR regions within human antibody frameworks, enabling the generation of high-affinity binders without the need for immunization (Griffiths et al., 1994). Fully synthetic libraries further expand this concept by introducing entirely designed sequences, allowing precise control over diversity and biophysical properties. Human VH and VL chains serve as the binding regions of antibodies. Artificially, VH and VL regions can be fused by a linker, forming a single-chain variable fragment (scFv). This scFv can be cloned into a vector next to PIII or PVIII, minor and major coat proteins, respectively (Weber et al., 2014). A natively paired VH: VL library was constructed from donor B cells using a microfluidic-based droplet platform. Individual B cells were co-encapsulated with oligo-dT beads and lysis buffer at a limiting dilution. Captured mRNA was re-encapsulated and recovered for reverse transcription and overlap extension PCR by amplifying the VH and VL regions using a gly-ser linker to form scFv (Zhou et al., 2021).

**FIGURE 2:**
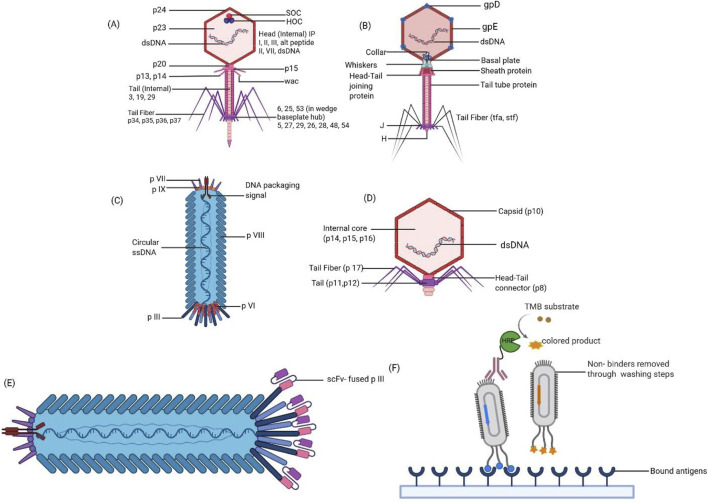
Structural organisation of various bacteriophages and phage display mechanism. **(A)** T4 bacteriophage-a contractile-tailed phage, tail fibres are involved in host injection, contains SOC and HOC, **(B)** λ phage-with a long, non-contractile tail, highlighting host attachment and DNA injection, **(C)** Filamentous phage M13-containing circular ssDNA provides a robust platform for peptide and antibody display, **(D)** T7 bacteriophage-with a short, non-contractile tail, internal core proteins facilitate genome delivery into the host cell, followed by adsorption through the tail fibres. **(E)** It highlights the M13 bacteriophage, where antibody fragments (scFv) are genetically fused to coat proteins such as pIII (minor coat protein), enabling their display on the phage surface. **(F)** The figure illustrates the selective binding of displayed antibodies to target antigens bound to the ELISA plate, followed by screening with anti-M13 HRP-conjugated antibodies to detect bound phages.

Usually, f1, fd, and M13 phages are used for expression and are transfected into *Escherichia coli* for the library generation. Figure 2 illustrates the structures of various phages used for antibody phage display, including T4, λ, M13, and T7. The M13 filamentous phage is highlighted as the most commonly used system for antibody display, where antibody fragments such as scFv are genetically fused to the pIII coat protein and displayed on the phage surface. It depicts antigen-specific binding followed by the detection of bound phages using anti-M13 HRP-conjugated antibodies. T7 bacteriophage is an alternative to M13 due to its robustness and stability against inactivation by other phages (Bazan et al., 2012). It can display smaller peptides (less than 50 residues) in higher copy numbers (Jaroszewicz et al., 2022). In addition to filamentous phages, phage lambda is used to express high-molecular-weight proteins. The lambda phage system elicits a stronger immune response than the filamentous phages. Also, this method does not require the transfer to bacteria (Bazan et al., 2012; Loganathan and Viswanathan, 2023).

Antibodies widely generated using phage display can be utilised for diagnostics and therapeutics. In 2002, adalimumab was the first clinically approved therapeutic antibody isolated by using phage display technology (Osbourn et al., 2005; Frenzel et al., 2016). Humira was developed against TNFα (tumour necrosis factor α) primarily to treat rheumatoid arthritis. The primary activity of adalimumab is its ability to bind to TNF-α, a pro-inflammatory cytokine. By binding TNF-α, adalimumab prevents TNF-α from interacting with its cell-surface receptors (TNFR1 and TNFR2) on various cells, thereby blocking downstream signalling pathways are involved in inflammation. It also prevented tissue destruction and other pathological processes associated with autoimmune diseases, such as rheumatoid arthritis, psoriatic arthritis, Crohn’s disease, and ulcerative colitis (Ellis and Azmat, 2025). Phage display technology has been widely applied for the isolation of disease-relevant antibodies. For example, it has been used to isolate a repertoire of human monoclonal anti-desmoglein autoantibodies from patients with active mucocutaneous pemphigus vulgaris (Payne et al., 2005).

#### 2.2.2 Bacterial display

Bacterial display technology was considered an alternative approach to phage display (Han et al., 2018). In contrast to phages, the expansive cell surface of *E. coli* allows for the selection and sorting of displayed libraries using fluorescence-based techniques and flow cytometric analysis of selected clones (Salema and Fernández, 2017). *Escherichia coli* is one of the most commonly employed host organisms for bacterial display (Salema and Fernández, 2017; Han et al., 2018). The Gram-negative bacterium has been explored extensively for research and development, mainly in biotechnology, owing to its rapid growth, well-characterised genetics, ease of manipulation, and cost-effective production (Salema and Fernández, 2017). A study by Shingarova, L. N., et al., presented an innovative approach to optimise the cell display technology for the 10th human fibronectin type III domain (10Fn3) scaffold protein, utilising the AT877 autotransporter from *Psychrobacter cryohalolentis* K5T. Using bacterial display techniques, the researchers engineered novel TNF-binding proteins by constructing and screening a combinatorial library of the 10Fn3 gene. Through the expression of selected 10Fn3 variants in *E. coli* cells, and analysing their TNF-binding capabilities, the study successfully identified proteins with significantly enhanced affinity for TNF (Shingarova et al., 2018). In Gram-negative bacterial display technology, selecting appropriate anchoring motifs is crucial for maintaining the stability of the cell envelope (Song et al., 2015). However, this is particularly challenging because proteins must traverse 2 cell membrane layers, complicating the display of larger proteins. In contrast, Gram-positive bacteria lack an outer membrane and possess a thick peptidoglycan cell wall; their single cell membrane facilitates the translocation of anchored fusion proteins (Rohde, 2019). Another study has used Gram-positive bacteria, *Staphylococcus* species, such as *Staphylococcus carnosus* and *Saccharomyces xylosus*, display proteins on their surface (Löfblom et al., 2017).

#### 2.2.3 Yeast display

The yeast display technology was developed back in 1997. It is a powerful tool for protein engineering, as it employs a eukaryotic host that dramatically enhances mammalian protein expression (Singh et al., 2023). *Saccharomyces cerevisiae, Pichia pastoris, Yarrowia lipolytica, and Hansenula polymorpha* are hosts for the display technology. This technique essentially displays the peptide or protein of interest on the surface of yeast. The protein of interest is generally positioned between epitope tags, such as the 9- amino acid Hemagglutinin tag (YPYDVPDYA) and a 10- amino acid C-Myc tag (EQKLISEEDL). These tags are further fused to the C- terminus of the Aga2P subunit, the anchor protein. This fused protein undergoes translation, and the 69-amino-acid Aga2P subunit joins the 725-amino-acid agglutinin Aga1P subunit through disulfide bonds. The resulting fused protein is secreted into the extracellular space, where Aga1P is covalently anchored to the cell wall, and the protein of interest is ultimately displayed on the yeast surface.

**FIGURE 3:**
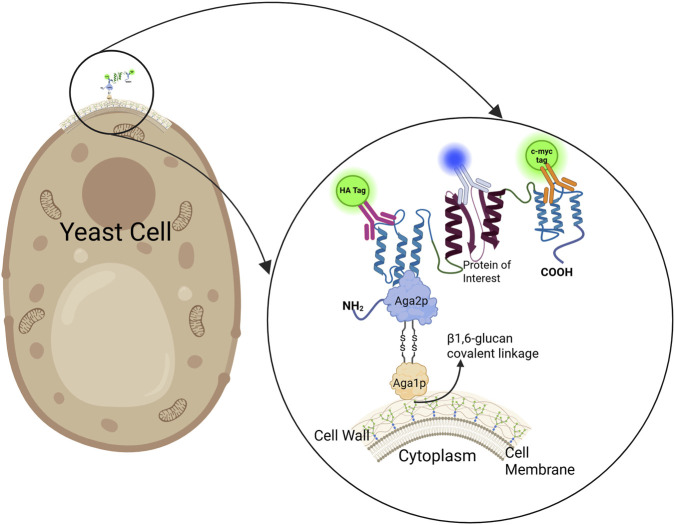
Yeast surface display using Aga1p-Aga2p anchoring system. Schematic representation of a *Saccharomyces cerevisiae* cell displaying a protein of interest on its surface. The protein of interest is fused to the Aga2p and transported through the secretory pathway. Aga2p has a disulfide linkage to Aga1p, which is anchored to the yeast cell through β-1,6 glucan linkages. Epitope tags, such as the HA tag, facilitate quantitative or semi-quantitative expression analysis, screening of binding affinity, by flow cytometry during library selection.

Figure 3 is a schematic representation of a *S. cerevisiae* cell displaying a protein of interest on its surface. The protein of interest is fused to Aga2p and secreted into the extracellular space. Aga2p has a disulfide linkage to Aga1p, which is anchored to the yeast cells through β-1,6 glucan linkages (Boder and Wittrup, 1997). Epitope tags, such as the HA tag, facilitate the monitoring of quantitative expression, screening of binding affinity, and expression analysis. The displayed protein can be screened or selected using various techniques, such as fluorescence-activated cell sorting (FACS) and magnetically activated cell sorting (MACS), where the target protein is labelled with magnetic beads or fluorescent dyes and were used for the isolation of selective binders (Cherf and Cochran, 2015; Mustafa et al., 2024). The main advantage of the yeast display technology is that it is a eukaryotic display system with well-functioning post-translational modification machinery. It can also synthesise multiple disulfide bonds. It is less time-consuming than the other eukaryotic display systems and requires less technical effort (Cherf and Cochran, 2015). Still, a few drawbacks to this display technique include the fact that the yeast glycosylation process differs from that of mammalian cells. The yeast display library size is limited to 10^9^ variants, whereas in phage display, it is about 10^11^ variants (Mahdavi et al., 2022).

Eptinezumab is a monoclonal antibody developed by Lundbeck Seattle Biopharmaceuticals in 2020, utilising *P. pastoris* as the host. This monoclonal antibody is used to treat migraines in adults, acting by blocking pain-conducting nerve signals via CGRP to the brain, thereby reducing pain (Datta et al., 2021). Recently, an antibody was selected from the antibody library to bind to the wild-type viral envelope protein domain III and neutralise the dengue virus (Puri et al., 2013). In this study, the researchers have developed and validated the approach of using a competitive yeast display library sorting to isolate epitope-specific antibodies from a naïve antibody library quickly (Puri et al., 2013).

#### 2.2.4 Ribosomal display

Ribosomal display is a robust, *in vitro* technology for protein production. It is a cell-free technique in which the newly synthesised mRNA and proteins bind to ribosomes (Cong et al., 2016). It promotes the coupling of individual nascent proteins, their phenotypes, to corresponding mRNA or genotypes by forming stable protein-ribosome-mRNA complexes (PRM) (Zahnd et al., 2007). The nascent proteins are linked to mRNA molecules via PRM complex formation in cell-free systems such as *E. coli* S30 or rabbit reticulocyte lysates. (3) Usually, extensive libraries are used for the selection procedure, and this technique integrates the *in vitro* amplification with the random mutagenesis (Zahnd et al., 2007; Dreier and Plückthun, 2018). In cell-free systems, the rabbit reticulocyte system contains DTT. This can affect folding stability, which differs from one antibody to another, possibly by altering intra-chain disulfide bridge formation. The presence of DTT affects the protein formation in prokaryotes, resulting in fewer functional antibody ribosome complexes. Also, 2–5 mM DTT was reportedly used for refolding the denatured proteins in a rabbit reticulocyte lysate system (Dreier and Plückthun, 2018).

#### 2.2.5 Mammalian cell display

**FIGURE 4:**
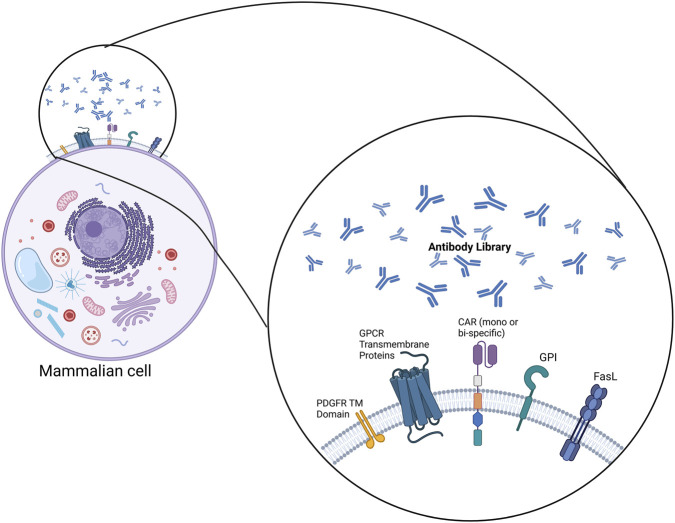
Mammalian cell-surface display representing a diverse antibody library. It illustrates various anchoring strategies for antibody libraries expressed on the surface of the plasma membrane, including transmembrane domains, PDGFR domains, GPI anchors, or CA architectures, as well as FasL. These anchoring proteins enable the stable surface expression of the antibody fragments for functional and affinity-based screening.

The rationale for developing the mammalian cell display platform was to enhance affinity maturation of scFv fragments against their targets (Robertson et al., 2020). Typically, the display is performed on the surface of human embryonic kidney 293T (HEK-293T) cells and Chinese hamster ovary (CHO) cells, using vectors from the pcDNA series, the pDGB vector, and a few other viral vectors. In this display technology, the expressed gene sequence generated by recombinant DNA technology is fused to the transmembrane (TM) sequence of a mammalian cell receptor. It then ultimately displays the overexpressed recombinant exogenous protein of interest on the surface of the mammalian cell. The fused TM sequence helps the expressed protein anchors to the cell surface (Ho and Pastan, 2009). Usually, PDGFR TM domains and GPCR TM domains help in the expression, as illustrated in Figure 4. A key advantage of mammalian display systems is their ability to express proteins in a physiologically relevant environment, enabling proper folding and native-like post-translational modifications, including N- and O-linked glycosylation. In addition, the use of eukaryotic expression machinery improves protein quality compared with yeast and phage display systems (Slavny et al., 2024). The major drawback of this display is its expense, time-consuming nature, and labour intensity (Jin et al., 2022).

Atezolizumab is designed to target the PD-L1 receptor and was approved by the FDA for multiple cancers, such as NSCLC, urothelial carcinoma, urothelial bladder cancer, and breast cancer. It was isolated from a phage display antibody library, and after selection, it was genetically engineered and expressed in Chinese Hamster Ovary (CHO) cells (Shah et al., 2017). A combination of atezolizumab and bevacizumab was used to treat unresectable or metastatic hepatocellular carcinoma, yielding better outcomes than previously approved small-molecule drugs, such as sorafenib (Finn et al., 2020). It is an example showing how antibodies, through display technologies, can contribute to cancer-related clinical research.

## 3 Translational bottlenecks faced by each display technology

### 3.1 Hybridoma technology

Hybridoma Technology has undoubtedly been a powerful source for generating monoclonal antibodies. During the use of this technology, researchers have faced several challenges; among these, the first and foremost one occurs due to the low frequency of antigen-specific B-cells in peripheral blood, which means that there might be only 1 in 10000 B-cells that have a target-specific action. Creating a library through hybridoma technology has become challenging (Smith et al., 2017). The next challenge arises after the fusion of B cells with the hybridoma cells, as fusion itself is a stressful process for a cell, and chemical agents such as PEG, when used, may lead to the formation of a polykaryon, which affects the stability and functionality of the hybridomas (Sa and Je, 2015).

### 3.2 Phage display technology

Preparing a naive library requires a very large library size, and the actual size can be limiting, potentially reducing the effectiveness of the target-specific action. Small libraries often fail to yield high-definition antibodies. The display level of single-chain variable antibody fragments can vary. This inconsistency in the expression can affect the efficiency of selection. On the other hand, a phage library can be prepared from immunised animals. However, the process of immunising the animal for antibody production and library development is a time-consuming process that limits the speed of developing therapeutic antibodies; the most significant challenge arises with the nature of antigens, whereas, in some cases, antigens are toxic and non-immunogenic, which obstructs the production of the library (Bazan et al., 2012).

### 3.3 Yeast display technology

During library generation, achieving both diversity and functional protein expression is critical. However, in yeast display systems, library diversity can be limited by PCR-based amplification, which may introduce biases. Additionally, the choice of fusion or anchoring protein for surface display can affect protein folding, thereby influencing stability and functional activity (Pepper et al., 2008).

### 3.4 Ribosomal display technology

The ribosomal system can form more extensive libraries than phage display or other cell-based systems, but developing a functional library with a high number of correctly folded proteins is a major challenge, as ribosomal display lacks the cellular machinery that helps fold proteins properly. In the absence of these machineries, misfolded proteins form, lacking functionality and diversity. Another challenge is stability. As we know, the ribosomal display system relies on creating a stable mRNA-ribosome protein. This complex can be affected by temperature, ionic strength, pH, and the concentrations of components involved in the display technology.

These unstable complexes lead to the loss of properly folded proteins (Kizerwetter et al., 2023).

### 3.5 Bacterial display technology

During library expansion, certain transformed cells may grow faster than others, leading to clonal dominance and a reduction in overall library diversity. Now, this process can reduce the variety of antibodies available for selection, which is a big challenge for researchers. The stability of the transformed cells is another topic of concern. Although bacterial systems are typically cultured at optimal growth temperatures (37 °C), improper handling or non-sterile conditions can lead to contamination, which adversely affects culture stability and antibody yield.

### 3.6 Mammalian cell display technology

One of the major challenges in mammalian display technology is constructing large-scale libraries and screening antibodies in their natural form against membrane proteins, as these proteins are difficult targets due to their complex structure or instability outside the cellular environment, making them very difficult to work with. This limits the type of target antibody to be screened. According to a previous study, the generation of a large-scale mammalian scFv library using lentiviral transduction of approximately 1.35 × 10^9^ CHO cells cultured in 1.5 L at a multiplicity of infection (MOI) of 1. The library was constructed using synthetic V(D)J segments to maximize sequence diversity. The theoretical diversity of mammalian display libraries can reach approximately 8 × 10^8^ variants; the effective diversity is often reduced to around 6 × 10^8^ due to limitations in transduction efficiency, expression levels, and clonal representation (Robertson et al., 2021). Another challenge is the incompatibility of the antibodies against the targets in their native conformation in the mammalian cells, which can result in sequence alterations and reduced antibody function. Since purified antigens must be presented to cells either in solution or immobilised to beads, target selection is largely restricted to soluble and structurally stable proteins and domains. This highlights the practical constraints on mammalian display (Robertson et al., 2021).

## 4 Role of glycosylation in maintaining antibody function and stability

Among all the PTMs in a cell, glycosylation has the most significant impact, as it can influence the structure, function, and stability of antibodies (Edwards et al., 2022). Antibodies, particularly IgG, bind to antigens at their target binding sites. Then, the Fc region of the antibody interacts with the Fc receptor, that is, the Fc gamma receptor (FcγR) on the surface of the effector cell, which is usually the natural killer cell. Natural killer (NK) cells recognise and bind to the Fc region of antibodies attached to target cells through Fcγ receptors, mediating antibody-dependent cellular cytotoxicity (ADCC) (Gómez Román et al., 2014; Lo Nigro et al., 2019). Another mechanism by which antibodies mediate cell destruction is complement-dependent cytotoxicity (CDC), where antibodies bind to the antigen-binding site on the surface of the target cells. The binding of the C1q protein binding to the antibody’s Fc region triggers a cascade of events. This leads to the formation of a membrane attack complex (MAC), leading to the destruction of the targeted cell. Glycosylation significantly influences the interaction between the Fc region of the antibody and the FcγR on the natural killer cell, which is responsible for ADCC and CDC (Gómez Román et al., 2014; Zahavi et al., 2018; Lo Nigro et al., 2019). Some glycoforms can either enhance or inhibit the interaction between the antibody and the Fc region of the effector cells, thus affecting the antibody’s ability to stimulate the production of immune effector cells. The N-linked glycan at Asn-162 of FcγRIIIa plays a critical role in Fc recognition, and removal of core fucose from IgG Fc glycans alleviates steric hindrance with this receptor-associated carbohydrate, thereby enhancing NK cell-mediated ADCC. However, while afucosylation can increase FcγRIIIa binding affinity and augments ADCC by approximately 2–40 fold in NK cell-based systems, the functional impact of Fc glycosylation is not universal and is critically dependent on the recruited effector cell type; additionally, antibody glycosylation also influences stability and solubility (Peipp et al., 2008; Golay et al., 2022). One study demonstrated that Fc afucosylation enhanced ADCC in a quantitatively, in a dose-dependent manner, without affecting antigen binding. By generating IgG4 antibodies with defined levels of afucosylation through blending of CHO- and Fut8-knockout CHO-derived cells, ADCC activity was observed to increase linearly with afucosylation levels, becoming detectable only roughly above 20% afucosylation and reaching maximal activity at 100% afucosylation (Gong et al., 2016). Glycosylation protects antibodies from proteolytic enzymes and prevents degradation. The glycan structure in the antibody makes it less prone to enzymatic cleavage and degradation. It also provides thermal stability to antibodies; glycosylated antibodies have been found to possess greater thermal stability, and glycans attached to antibodies alter their hydrodynamic radius, thereby enhancing solubility (Zheng et al., 2011). It stabilises the Fc region, which is crucial for binding to effector cells and maintaining a proper conformation (Edwards et al., 2022).

## 5 Sequencing-enabled emerging technologies in antibody discovery

In addition to the display technologies, single-cell RNA sequencing (scRNA-seq) serves as a transversal measurement and profiling technology that enables the identification of antibody gene pairings, immune repertoire analysis, and the elucidation of disease heterogeneity. Single-cell technology research began in 2009, when F. Tang, Y. Wang, et al. described the whole transcriptome of a single cell.

This technique also plays a vital role in multi-omics and spatial transcriptomics of cells, enabling the discovery of cells at multiple levels and the identification of their locations, biomarkers,and co-expression patterns (Tang et al., 2009). In past years, a considerable amount of data has have been collected from projects like the Human Cell Atlas, the Mouse Cell Atlas, the Mouse RNA Atlas, and the Plant RNA Atlas, and based on these data, along with the help of artificial intelligence and bioinformatic algorithms for different cell-cell interactions, cell-cell transitions, cell type annotation, which means cell type identification and developmental inference have been described (Tang et al., 2009).

Bacteriologists have also adopted single-cell technology in microbiology research to study the growth phase, cell organisation, and cell division (Brehm-Stecher and Johnson, 2004). The first steps in single-cell sequencing involve single-cell isolation using various methods, such as FACS, micromanipulation, laser-captured microdissection (LCM), the ICELL8 system, or microfluidic platforms like the FLUIDIGM C1 system. Afterwards, the whole genomic DNA from the isolated cell is amplified, followed by purification of the amplified product, which is then assessed for concentration, size, and uniformity. Finally, sequencing libraries are constructed and analysed using next-generation sequencing (NGS). DNA sequencing can be performed using various techniques, including Degenerate Oligonucleotide Priming-Polymerase Chain Reaction (DOP-PCR), Multiple Displacement Amplification (MDA), and Multiple Annealing and Looping-based amplification cycle (MALBAC). Single-cell RNA sequencing can also be performed using methods such as SMART-seq, SMART-seq2, CEL-seq, etc (Shen, 2019). Various computational methods have also been developed to analyse data, including Cutadapt, FastQC, BWA, Picard, ReadCount, Monovar statistical model, IDBA-UD, and others (Shen, 2019). Single-cell technology has been a great support in cancer research. For example, the scientists from the Institut Curie had collected tumour cells from one of their patients who was non-responsive to the drug. The collected cells were studied in a microfluidic device and then encapsulated in an oil droplet containing reagents. During encapsulation, the genetic material of each cell is labelled with a unique barcode that enables cell-specific identification. Then, the sample is sent for sequencing, and the genetic data is read using bioinformatic tools (CNRS News, 2017). Cancer studies at the granular level are widely enhanced, particularly with the help of scRNA-seq. Detection of rare cell populations within tumours and the tumour microenvironment, along with the identification of specific biomarkers, enables the development of personalised treatment strategies for cancers characterised by intra-tumoral heterogeneity (Smith and Zauderer, 2014). ScRNA-seq aids in patient stratification by resolving tumour heterogeneity through the identification of gene expression profiles, gene mutations, immune checkpoints, antigenic phenotypes, and cellular states (Ying et al., 2020).

The development of computational methods that utilise scRNA sequencing data to associate individual cells with diseases also plays a significant role in diagnosing and personalizing therapy. One study demonstrated that scRNA-seq analysis facilitated the identification of CAR targets for genes highly expressed in tumours by screening both malignant and normal cells at the single-cell level. They have also incorporated machine learning algorithms, such as Random Forest (RF) and a convolutional neural network (CNN), for processing single-cell transcriptomes (Kwon et al., 2023). Thus, in short, single-cell technology, particularly through scRNA-seq can provide pathways to more personalised cancer therapies by enabling the study of tumour heterogeneity and the identification of specific biomarkers (Nath and Bild, 2021). In another study, the authors developed a method for Linking B-cell Receptor to Antigen (LIBRA) specificity through next-generation sequencing (NGS). In this method, the DNA-barcoded antigens were mixed with the donor B cells, and then the antigens bound to specific B cells were recovered and sequenced simultaneously with the binding BCRs. This high-throughput approach enabled the identification of antigen-specific antibodies from patients, which would have been complex to identify and sort otherwise (Setliff et al., 2019). It has previously been shown that pathogen-specific and virus-neutralising antibodies can be identified in the patient serum without determining the antibody sequence. Antigen-expressing phages were used to identify the immunoglobulin-expressing cells using FACS sorting. This enables the identification of antigen-specific antibody enrichment without the need for individual antibody expression and purification. Thus, the approach saves both time and cost, enabling antibody development within a few weeks (Lomakin et al., 2024).

Additionally, DNA-encoded libraries (DELs) facilitate epitope discovery and mapping. They are composed of synthetically generated small-molecule chemotypes, each covalently coupled to a DNA tag that encodes its structure, enabling simultaneous synthesis and selection, unlike traditional high-throughput sequencing (HTS). This makes DELs accessible and affordable. Previously, DELs were selected against immobilised target proteins, while washing off the non-binders. Several methods have been recently developed for selecting DNA-encoded small-molecule libraries against non-immobilised protein targets. One of these methods involves directly selecting DELs for endogenous proteins in cell lysates. Today, DELs can be created as vast libraries of small molecules, where each compound carries a unique DNA tag as its identity label. After screening, high-affinity binders can be easily identified by reading these DNA tags, thus eliminating the need for chemical isolation and characterisation (Zhao et al., 2014).

## 6 Integration of AI in revolutionising drug discovery and antibody development

The traditional approaches to antibody development are cumbersome and time-consuming. The approach of injecting mice with antigens to raise antibodies is challenging, as the antibodies need to be further humanised to avoid adverse drug reactions. Antibody production using display technologies also requires significant time and wet-lab procedures. Also, a considerable challenge in antibody discovery is the aggregation of the molecule and non-specific binding. Several studies have reported the *de novo* design of antibody libraries using computational tools integrated with AI and ML. These antibodies are specifically designed to target particular epitopes; hence, the overall efficacy of the antigen-antibody interaction increases. Antibody development, specifically when integrated with machine learning tools, is applied to improve humanisation, better predict epitope binding, generate sequence libraries, and, finally, predict antigen-antibody interactions (Matsunaga and Tsumoto, 2025). Double-blind assessments have shown that the Rosetta algorithm is one of the most successful methods for *de novo* protein structure prediction (Rohl et al., 2004). It employs a Monte Carlo-based approach to assemble short fragments of known protein structures into native-like conformations. Rationally designing an antibody requires a critical understanding of the variable fragment structure, which includes six hypervariable loops of complementarity-determining regions (CDRs) (Ruffolo et al., 2022). DeepAb is a tool comprising two phases: the first, in which the network predicts the variable fragment (Fv) structure based on the input light and heavy chain sequences; and the second, which utilises Rosetta-based methods to predict structures from the network (Yu et al., 2024). AlphaFold (AF) and its versions, AF3, developed by Google DeepMind, are machine learning algorithms for the 3D-structural prediction of proteins, modelling predicted structures, and computational protein design (Yang et al., 2023). Although computational approaches for protein modelling look promising, their application in antibody discovery remains challenging. The simulations are computationally intensive and often fail to capture the context-dependent nature of protein interactions. While evolutionary and deep learning-based approaches have improved structural predictions, their accuracy remains limited, particularly in the absence of closely related templates, necessitating continued experimental validation (Jumper et al., 2021).

**FIGURE 5:**
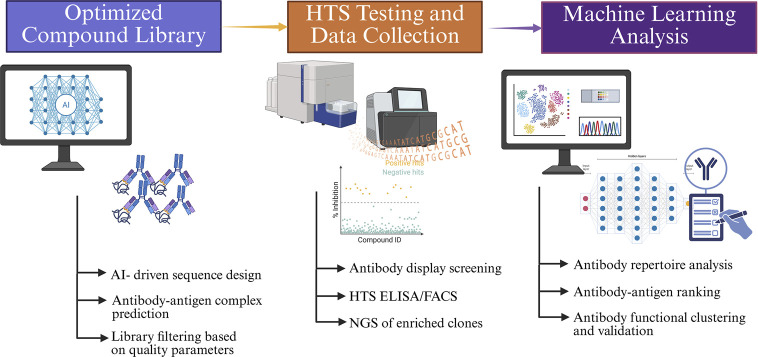
AI-assisted rational antibody design and optimisation pipeline. It illustrates an integrated workflow combining computational and experimental approaches for next-generation antibody discovery. Initial *in silico* library pre-optimisation is performed using structural modelling, antibody-antigen complex prediction, and protein language model-based sequence generation to reduce library size while maximising functional diversity. These computationally prioritised libraries are subsequently expressed and interrogated using HTS platforms, including display-based selection, binding assays, and functional readouts such as neutralisation or effector activity. Data generated from HTS are then fed into machine-learning (ML) models to enable multidimensional functional profiling, affinity, specificity, developability, and effector function predictions, as well as iterative refinement of sequence.

Figure 5 shows the AI-assisted rational antibody design and optimisation pipeline. It illustratesan integrated workflow combining computational and experimental approaches for next-generation antibody discovery. Initial *in silico* library pre-optimisation is performed using structural modelling, antibody-antigen complex prediction, and protein language model-based sequence generation to reduce library size while maximising functional diversity. These computationally prioritised libraries are subsequently expressed and interrogated using HTS platforms, including display-based selection, binding assays, and functional readouts such as neutralisation or effector activity. Data generated from HTS is then fed into ML models to enable multidimensional functional profiling, affinity, specificity, developability, and effector function predictions, as well as iterative refinement of sequence space.

## 7 Conclusion and future perspectives

Antibody display technologies have revolutionised the discovery, optimisation, and engineering of therapeutic antibodies. Initially, from hybridoma technology to sophisticated display systems such as phage, bacterial, yeast, mammalian, and ribosomal platforms, each method has offered unique advantages in terms of affinity maturation, expression fidelity, library diversity, and ease of screening as the field has advanced. Integrating display technologies with single-cell omics and computational biology has advanced the antibody discovery pipeline into a more personalised, faster, and addresses a larger space of therapeutic targets. Despite technological advances, these methods have limitations, including a limited library diversity, expression challenges, scalability issues, and production costs, particularly when translating display-derived hits into biologics. However, technological advances, such as the development of synthetic scaffolds, enhanced host systems, and microfluidic platforms, are actively addressing these limitations in this area. The convergence of AI and deep learning algorithms, with traditional antibody engineering, is likely to redefine the therapeutic antibody landscape. AI- based models such as AlphaFold, DeepAb, and Rosetta enable accurate structural predictions and epitope mapping, thereby significantly accelerating lead candidate optimisation. Furthermore, the integration of machine learning with high-throughput screening platforms and the emergence of AI-powered wet labs paved the way for the design of next-generation, patient-specific antibody therapeutics in a fraction of the time required in traditional development. The future of antibody discovery lies in creating flexible platforms that enable faster lead generation and on-demand production of biologics tailored to individual patient profiles. With innovations in single-cell sequencing, transcriptomics, and data analysis, researchers can dissect disease heterogeneity and design more precise, stable, and effective antibodies.

In conclusion, we suggest that continued investment in interdisciplinary innovation, spanning molecular biology, immunology, computational modelling, and antibody engineering, is critical to unlock the full therapeutic potential of display technologies. As we move towards precision and personalised medicine, antibody display technologies will lead the biologics discovery, bridging the gap between benchside innovation and bedside application. By highlighting both technological advancements and current limitations, this review provides a forward-looking framework for improving antibody engineering and therapeutic development.
